# Neurological prognostication after cardiac arrest: how the “Best CPC” project would overcome selection biases

**DOI:** 10.1186/s13054-019-2533-3

**Published:** 2019-07-05

**Authors:** Marine Paul, Stéphane Legriel

**Affiliations:** 1 Medical-Surgical Intensive Care Department, Centre Hospitalier de Versailles - Site André Mignot 177 Rue de Versailles, 78150 Le Chesnay, France; 2 AfterROSC Study Group, Paris, France; 3 Sorbonne Paris Cité-Medical School, Paris Descartes University, Paris, France; 4 Cardiovascular Research Center, INSERM U970, Paris, France

## Background

Despite perpetual improvement in post cardiac arrest (CA) care, a substantial number of patients with CA remain comatose after rewarming from targeted temperature management (TTM) and sedation discontinuation. Many patients in this situation eventually die, usually after withdrawal of life-sustaining treatment (WLST) due to a poor neurological prognosis [1, 2]. Neuroprognostication after cardiac arrest relies on a multimodal approach in which clinical examination, neurophysiological tools (electroencephalogram, somatosensory evoked potentials), biomarkers (neuron-specific enolase and S-100β) and brain imaging (computed tomography scan and magnetic resonance imaging) can be used in various combination for which the input mode is a confirmed postanoxic comatose state [3, 4].

However, these clinical and ancillary tests recommended in guidelines for neuroprognostication after CA were chiefly validated in retrospective studies of heterogeneous patient populations [5] potentially responsible for selection biases.

## Main text

Neuroprognostication in post-anoxic coma aims chiefly to identify those patients likely to have a poor neurological prognosis, i.e., a Cerebral Performance Category (CPC) score of 3 to 5 or a modified Rankin Scale score of 3 to 6, as opposed to a CPC score of 1–2 or a modified Rankin Scale score of 0 to 2 [6]. However, there is an important heterogeneity in these patients. Indeed, CPC scores of 3 and 4 are straightforward, as they indicate neurologic impairments due to anoxia. A CPC score of 5, i.e., death, in contrast, is not necessarily related directly to irreversible brain damage from anoxic encephalopathy. Other causes of death include post-cardiac arrest syndrome with multi-organ failure, acute respiratory distress syndrome, sepsis, high comorbidities leading to treatment-limitation decisions and recurrent CA with or without awakening. Failure to differentiate these non-neurological causes of death may result in major interpretation bias studies focused on neurological prognostication, leading to inappropriate conclusions about the predictive value of neuroprognostic tests (e.g. electroencephalogram findings) and about the impact of treatments on neurological outcomes.

Optimal neuroprognostication following CA would be performed, if it were possible, in a homogeneous population of patients remaining comatose after TTM management and after sedation was discontinued. Predictors of unfavourable outcome could be defined in such a population sub-set. Indeed, current guidelines recommend initiating neurological prognostication in patients with persistent coma 72 h after the CA, with the intent of not including those subsets of patients who will die early of multi-organ failure, or those that rapidly progress towards brain death. In an ancillary study of a TTM trial [7], Dragancea et al. found that the reason for WLST was neurological impairments in 85% of patients who required prognostication [8]. However, 18% of WLST decisions were based on ethical reasons or severe comorbidities. Recently Witten et al. suggested classifying causes of death after CA as WLST for neurological reasons, WLST due to comorbidities, refractory shock, recurrent CA and respiratory failure [9]. Among patients who died after out-of-hospital CA, 73% had their life-support withdrawn for neurological reasons. Inter-rater variability in the assessment of causes of death was noted, and some patients had more than one cause of death, chiefly for life-support withdrawal due to comorbidities or haemodynamic impairment. The broad range of conditions in comatose patients after CA may have resulted in biases in the development of the currently used neuroprognostication tools. Consequently, to improve neuroprognostication, predicting unfavourable neurological outcome should be limited to patients persistent in a comatose state beyond 72 h, a consequence of arrest-induced hypoxia-ischemia.

Conversely, predicting a favourable neurological outcome after CA would involve predicting the ability to awaken independent of final outcome, even if the patient secondarily dies. Patients with non-neurological aetiology of death prior to weaning off sedation that precludes examination of arousal should not be included in such studies either. The best daily CPC score proposed by Taccone et al. may help to assess predictors of awakening and a significant rate of late awakeners was recently reported [10–12]. Furthermore, in the study by Taccone et al., standard neuroprognostication criteria including electroencephalogram findings, somatosensory evoked potentials, and neurospecific enolase levels were not associated with poor neurological outcomes in patients with death after awakening [13]. According to this strategy, the prediction of a favourable outcome would allow identifying patients that will awaken in the ICU after cardiac arrest. This category of patients who awaken would henceforth be managed as any other critical care patient and be subject to the same ICU complications and medical outcomes, as well as be subject to the discussions of end-of-life and withdrawal of care as dictated by clinical course and family considerations. Thus, in case of favourable neurological outcome prediction, we propose to focus the problem in evaluating the ability of awakening regardless of final survival status.

We recognise the limitations to our proposals. Firstly, our strategy of focusing the metric upon the favourable measure of awakening regardless of ultimate outcome will carry a bias in the over-representation of predicted final survivors, but the dividend would be that such evaluation might improve diagnostic accuracy of neuroprognostication tools. Secondly, we fully acknowledge that our proposals require confirmation via formal prospective evaluation before the “BEST CPC” concept can make a positive impact on post CA clinical care.

## Conclusion

In conclusion, currently used neuroprognostication tools may lack diagnostic accuracy because they were developed and validated in heterogeneous populations of patients, including patients who remained continuously comatose, patients who awakened before dying, and patients erroneously classified as having died of neurological causes. Focusing on awakening and thus using the best daily CPC score may prove helpful for assessing neuroprognostication tools intended for comatose patients after CA.

## Data Availability

Not applicable.

## Abbreviations

CA: Cardiac arrest
CPC: Cerebral Performance Category
DAA: Death after awakening
TTM: Targeted temperature management
WLST: Withdrawal of life-sustaining treatment
